# Internalization of the Active Subunit of the *Aggregatibacter actinomycetemcomitans* Cytolethal Distending Toxin Is Dependent upon Cellugyrin (Synaptogyrin 2), a Host Cell Non-Neuronal Paralog of the Synaptic Vesicle Protein, Synaptogyrin 1

**DOI:** 10.3389/fcimb.2017.00469

**Published:** 2017-11-14

**Authors:** Kathleen Boesze-Battaglia, Lisa P. Walker, Anuradha Dhingra, Konstantin Kandror, Hsin-Yao Tang, Bruce J. Shenker

**Affiliations:** ^1^Department of Biochemistry, School of Dental Medicine, University of Pennsylvania, Philadelphia, PA, United States; ^2^Department of Pathology, School of Dental Medicine, University of Pennsylvania, Philadelphia, PA, United States; ^3^Department of Biochemistry, School of Medicine, Boston University, Boston, MA, United States; ^4^Wistar Proteomics and Metabolomics Core Facility, Wistar Institute, Philadelphia, PA, United States

**Keywords:** lymphocytes, toxin, cytolethal distending toxin, *Aggregatibacter actinomycetemcomitans*, pathogenesis, cell cycle arrest, apoptosis

## Abstract

The *Aggregatibacter actinomycetemcomitans* cytolethal distending toxin (Cdt) is a heterotrimeric AB_2_ toxin capable of inducing lymphocytes, and other cell types, to undergo cell cycle arrest and apoptosis. Exposure to Cdt results in binding to the cell surface followed by internalization and translocation of the active subunit, CdtB, to intracellular compartments. These events are dependent upon toxin binding to cholesterol in the context of lipid rich membrane microdomains often referred to as lipid rafts. We now demonstrate that, in addition to binding to the plasma membrane of lymphocytes, another early and critical event initiated by Cdt is the translocation of the host cell protein, cellugyrin (synaptogyrin-2) to the same cholesterol-rich microdomains. Furthermore, we demonstrate that cellugyrin is an intracellular binding partner for CdtB as demonstrated by immunoprecipitation. Using CRISPR/cas9 gene editing we established a Jurkat cell line deficient in cellugyrin expression (Jurkat^Cg−^); these cells were capable of binding Cdt, but unable to internalize CdtB. Furthermore, Jurkat^Cg−^ cells were not susceptible to Cdt-induced toxicity; these cells failed to exhibit blockade of the PI-3K signaling pathway, cell cycle arrest or cell death. We propose that cellugyrin plays a critical role in the internalization and translocation of CdtB to critical intracellular target sites. These studies provide critical new insight into the mechanism by which Cdt, and in particular, CdtB is able to induce toxicity.

## Introduction

Intracellular-acting microbial toxins face the common need to gain entry into the cytosol of host target cells. To meet this challenge, microbes have developed numerous strategies for toxin internalization as well as mechanism(s) by which they incapacitate cells (Oswald et al., 2005; Donaldson and Williams, 2009; Lebrun et al., 2009). Once inside, bacterial toxins typically hijack existing endocytic trafficking pathways to deliver their active component to appropriate subcellular targets. It is becoming increasingly clear that one bacterial toxin, the cytolethal distending toxin (Cdt), produced by the oral pathogen *Aggregatibacter actinomycetemcomtans* and over 30 γ- and ε- Proteobacteria, has developed a unique approach to overcoming these common challenges (Boesze-Battaglia et al., 2016; Scuron et al., 2016). The *A. actinomycetemcomitans* Cdt is a heterotrimeric complex consisting of three subunits designated CdtA, CdtB, and Cdt C which collectively function as an AB_2_ toxin (de Rycke and Oswald, 2001; Elwell et al., 2001; Lara-Tejero and Galan, 2001; Nesic et al., 2004; Shenker et al., 2004, 2005; Thelestam and Frisan, 2004; Gargi et al., 2012). The first step leading to cell intoxication requires that Cdt binds to cell surfaces; this occurs via the cell binding unit (B) consisting of subunits CdtA and CdtC reviewed in Boesze-Battaglia (2006) and Gargi et al. (2012). This complex is responsible for not only toxin binding to the cell surface but also subsequent delivery of the active subunit (A), CdtB, to intracellular compartments. The exact role for CdtA in binding to cells is not clear, but several studies have suggested that this subunit may recognize a range of targets including fucose moieties and glycosphoingolipids, among others (Nesic et al., 2004; Mise et al., 2005). It should also be noted the Cdt binding occurs in the context of cholesterol/sphingomyelin-rich membrane microdomains, commonly referred to as lipid rafts. This association is the result of the CdtC subunit's ability to recognize and bind to cholesterol via cholesterol recognition sequences known as CRAC sites (Guerra et al., 2005; Boesze-Battaglia et al., 2009, 2015; Eshraghi et al., 2010; Zhou et al., 2012; Lai et al., 2013). These observations are particularly significant as membrane cholesterol rich microdomains have been shown to serve a number of relevant functions including concentrating toxins on the cell surface and providing access to molecular pathways associated with endocytosis and signaling (Cherukuri et al., 2001; Dykstra et al., 2003).

The mechanism by which CdtB induces toxicity is controversial and has recently been reviewed (Scuron et al., 2016). Briefly, we have demonstrated that CdtB functions as a lipid phosphatase capable of degrading the signaling lipid, phosphatidylinositol-3, 4, 5-triphosphate (PIP3), thereby causing PI-3K signaling blockade and conditions that trigger cell cycle arrest and apoptosis. Other investigators propose that CdtB function as a DNase capable of causing DNA strand breaks which in turn lead to toxicity (Elwell and Dreyfus, 2000; Cortes-Bratti et al., 2001; Frisan et al., 2002; Nesic et al., 2004; Thelestam and Frisan, 2004). Nonetheless, internalization of CdtB has been shown to be essential for the induction of toxicity. CdtB internalization has been shown to involve cholesterol recognition as well as endocytic mechanisms that are dynamin dependent and which involve clathrin coated pits (Cortes-Bratti et al., 2000; Thelestam and Frisan, 2004; Boesze-Battaglia et al., 2009, 2015; Guerra et al., 2011). However, there is controversy as to how this active subunit is transported through the cell cytosol. Some studies suggest a role for the ER-associated degradation (ERAD) pathway, while others have failed to demonstrate ERAD involvement (Guerra et al., 2009; Eshraghi et al., 2014). We now report that immunoprecipitation and proteomic analysis of cell extracts derived from Jurkat cells treated with *A. actinomycetemcomitans* Cdt identified a novel protein, cellugyrin, that associates with CdtB complexes. Furthermore, we demonstrate that internalization of *A. actinomycetemcomitans* CdtB is dependent upon cellugyrin, also known as synaptogyrin 2 (Kedra et al., 1998). These findings are consistent with those of Carette et al. (2011) who suggested a link between Cdt intoxication and cellugyrin. Cellugyrin is a tetraspanin membrane protein expressed in most cells and is a non-neuronal paralog of the synaptic vesicle protein, synaptogyrin 1 (Janz and Sudhof, 1998). Moreover, it is proposed to be a component of ubiquitous intracellular transport vesicles that mediate protein transport between sorting endosomes and the endocytic recycling compartment and/or trans-Golgi network (TGN) (Kupriyanova and Kandror, 2000).

In this paper we demonstrate that in concert with Cdt binding to cholesterol and association with membrane microdomains, cellugyrin levels increase and the protein translocates from the cytoplasm to cholesterol rich membrane microdomains. We further demonstrate that cellugyrin is a critical component of an intracellular complex that binds to CdtB; this interaction is critical to CdtB internalization. Moreover, we demonstrate that cells deficient in cellugyrin are unable to internalize CdtB and they are also resistant to its intoxication.

## Materials and methods

### Reagents and antibodies

Construction and expression of the plasmid containing the *cdt* genes for the holotoxin (pUCAacdtABC^his^) has previously been reported (Shenker et al., 2004). The histidine-tagged peptide holotoxin was isolated by nickel affinity chromatograpy as previously described (Shenker et al., 2000). Murine monoclonal antibodies to Cdt subunits were prepared as previously reported (Boesze-Battaglia et al., 2006). Rabbit polyclonal antibody against a cellugyrin peptide (Ac-CQNVETTEGYQPPPVY-OH) was raised and affinity purified (Kim and Kandror, 2012). All other antibodies were obtained from commercial sources as indicated.

### LC-MS/MS analyses and data processing

Liquid chromatography tandem mass spectrometry (LC-MS/MS) analysis was performed by the Proteomics and Metabolomics Facility at the Wistar Institute using a Q Exactive HF mass spectrometer (ThermoFisher Scientific; Waltham, MA) coupled with a Nano-ACQUITY UPLC system (Waters; Milford, MA). Samples were digested in-gel with trypsin and injected onto a UPLC Symmetry trap column (180 μm i.d. × 2 cm packed with 5 μm C18 resin; Waters). Tryptic peptides were separated by reversed phase HPLC on a BEH C18 nanocapillary analytical column (75 μm i.d. × 25 cm, 1.7 μm particle size; Waters) using a 95 min gradient formed by solvent A (0.1% formic acid in water) and solvent B (0.1% formic acid in acetonitrile). A 30-min blank gradient was run between sample injections to minimize carryover. Eluted peptides were analyzed by the mass spectrometer set to repetitively scan m/z from 400 to 2,000 in positive ion mode. The full MS scan was collected at 60,000 resolution followed by data-dependent MS/MS scans at 150,000 resolution on the 20 most abundant ions exceeding a minimum threshold of 20,000. Peptide match was set as preferred, exclude isotopes option and charge-state screening were enabled to reject unassigned charged ions.

Peptide sequences were identified using MaxQuant 1.5.2.8 (Cox and Mann, 2008). MS/MS spectra were searched against a custom UniProt human protein database containing cytolethal distending toxin sequences using full tryptic specificity with up to two missed cleavages, static carboxamidomethylation of Cys, and variable oxidation of Met, and protein N-terminal acetylation. Consensus identification lists were generated with false discovery rates of 1% at protein and peptide levels.

### CRISPR/cas9 mediated genome editing

CRISPR/cas9 technology was employed to generate cellugyrin knockout Jurkat cells (Jurkat^Cg−^) using commercially available reagents (Santa Cruz Biotechnology; Santa Cruz, CA). The Amaxa Nucleofector system (Lonza; Basel) was used to transfect cells with a pool of three plasmids each encoding the Cas9 nuclease and a cellugyrin-specific 20 nt guide RNA (gRNA). Cells were co-transfected with a pool of three plasmids each containing a homology-directed DNA repair (HDR) template corresponding to sites generated by the cellugyrin CRISPR/cas9 knockout plasmid. The HDR plasmids insert the puromycin resistance gene for selection of stable knockout cells. Cells were incubated 5 days after transfection followed by incubation in puromycin (5 μg/ml) for an additional 7 days. Surviving cells were cloned by limiting dilution. Clones were expanded and evaluated by Western blot analysis for the presence of cellugyrin; clones deficient in cellugyrin were cloned a second time by limiting dilution. Cells were maintained in medium containing puromycin (1 μg/ml).

### Cell culture, cell cycle, and apoptosis

The T cell leukemia cell line Jurkat [E6-1; (American Type Tissue Culture Collection; Manassas, VA)] was maintained as previously described (Shenker et al., 2004). Briefly, Jurkat^WT^ cells were maintained in RPMI 1640 supplemented with 10% FCS, 2 mM glutamine, 10 mM HEPES, 100 U/ml penicillin, and 100 μg/ml streptomycin. Jurkat^Cg−^ cells were maintained in the same medium with the addition of puromycin as described above. Human peripheral blood mononuclear cells lymphocytes (HPBMC) were prepared and incubated as described previously (Shenker et al., 1982). HeLa cells (CRL-1958; ATCC) were cultured in MEM supplemented with 10% fetal bovine serum and 2% antibiotic solution at 37°C in 5% CO_2_ (Shenker et al., 2016a).

To measure Cdt-induced cell cycle arrest, Jurkat^WT^ and Jurkat^Cg−^ cells were incubated in the presence of medium or Cdt for 16 h as previously described (Shenker et al., 2000). Briefly, cells were then washed and fixed for 60 min with cold 80% ethanol. After washing, the cells were stained with 10 μg/ml propidium iodide containing 1 mg/ml RNase (Sigma Chemical Co; St. Louis, MO) for 30 min. Samples were analyzed on a Becton-Dickinson LSR II flow cytometer (BD Biosciences; San Jose, CA). Propidium iodide fluorescence was excited by an argon laser operating at 488 nm and fluorescence measured with a 630/22 nm bandpass filter using linear amplification. A minimum of 15,000 events were collected on each sample; cell cycle analysis was performed using Modfit (Verity Software House; Topsham, ME).

To measure Cdt-induced apoptosis, Jurkat^WT^ and Jurkat^Cg^ cells were incubated for 48 h in the presence of medium or Cdt. DNA fragmentation was measured using the TUNEL assay [*In Situ* Cell Death Detection Kit; (Boehringer Mannheim)] (Shenker et al., 2001). Cells were harvested and re-suspended in freshly prepared 4% formaldehyde and permeabilized with 0.1% Triton X-100 for 2 min at 4°C. The cells were then washed with PBS and incubated in a solution containing FITC labeled nucleotide and terminal deoxynucleotidyl transferase (TdT) according to the manufacturers specifications. FITC fluorescence was assessed by flow cytometry using an argon laser at 488 nm to excite the fluorochrome; emission was measured through a 530/30 nm bandpass filter.

### Isolation of Triton X-100 resistant membrane rafts

Triton X-100 resistant cholesterol rich membrane microdomains were prepared from Jurkat cells using a previously published protocol (Boesze-Battaglia et al., 2006). Briefly, cell homogenates were centrifuged on a sucrose gradient at 40,000 rpm for 20 h at 4°C. Two prominent zones were recovered, designated DRM1 and DRM2; these were washed and resuspended in HEPES buffer. As previously reported, the lipid and protein composition of these zones were analyzed to verify that they were indeed rafts (Boesze-Battaglia et al., 2006).

### Immunoprecipitation and western blot analysis

Cells were treated as described and solubilized in 20 mM Tris-HCl buffer (pH7.5) containing 150 mM NaCl, 1 mM EDTA, 1% NP-40, 1% sodium deoxycholate, and protease inhibitor cocktail (ThermoFisher Scientific; Waltham, MA). Samples (30 μg) were separated on 12% SDS-PAGE and then transferred to PVDF membranes. The membrane was blocked with BLOTTO and then incubated with one of the following primary antibodies for 18 h at 4°C (Shenker et al., 1999): anti-Akt, anti-pAkt (S473), anti-GSK3β, anti-pGSK3β (S9), or anti-GAPDH (Cell Signaling Technology; Danvers, MA). Membranes were washed and incubated with goat anti-mouse immunoglobulin conjugated to horseradish peroxidase (Southern Biotech Technology; Birmingham, AL). The Western blots were developed using chemiluminescence and analyzed by digital densitometry (Li Cor Biosciences; Lincoln, NE) as previously described (Shenker et al., 2010).

For immunoprecipitation studies, control IgG or antibody to cellugyrin or CdtB was immobilized by crosslinking to protein A/G using the Pierce Crosslink IP kit (ThermoFisher Scientific). Jurkat cells, HPBMC, or HeLa cells were incubated with medium or Cdt (2 μg/ml) for 2 h at 37°C and then lysed and centrifuged at 5,000 × g for 5 min. Supernatants were loaded onto columns containing the protein A/G with crosslinked antibody (or control IgG) and incubated overnight at 4°C. The columns were washed and immunopreciptated protein eluted and analyzed by Western blot as described above. Western blots were developed using anti-cellugyrin antibody or anti-Cdt subunit mAb as described above.

### Immunofluorescence microscopy and flow cytometry

Jurkat cells were incubated with or without Cdt holotoxin (1 μg/ml) for 1 h at 37°C, washed twice, and re-suspended in RPMI 1640. Membrane lipid raft staining was performed by incubating the cells with 1 μg/ml cholera toxin B (CTB) conjugated to AlexaFluor 594 (Invitrogen, Carlsbad, CA) for 30 min at 4°C. The cells were washed in RPMI 1640, then incubated with mouse anti-CTB antibody (Invitrogen) at 4°C for 30 min followed by 37°C for 30 min, washed and fixed in cold methanol (−20°C) for 15 min. For cellugyrin staining, cells were blocked in 5% BSA and permeabilized with 0.2% Triton-X100 in PBS (PBST), incubated with rabbit anti-cellugyrin conjugated to Alexa Fluor 488 at 37°C for 1 h and washed in PBST. Additionally, cells were stained with Hoechst 33258 (AnaSpec Inc; Freemont, CA). Samples were mounted in Cytoseal mounting medium (Electron Microscopy Sciences, Hatfield, PA) and images captured with a Nikon A1R laser scanning confocal microscope with a PLAN APO VC 60 × water (NA 1.2) objective at 18°C. Image z-stacks were acquired at an interval of 0.3 μm (11 focal planes/image stack). Data were analyzed using Nikon Elements AR 4.30.01 software; for co-distribution analyzes, the Pearson's' coefficient was at least 0.55, and analysis included maximum intensity projection and standard LUT adjustment (Reyes-Reveles et al., 2017).

Cdt binding and internalization was detected by incubating Jurkat cells for 30 min (surface staining) or 1 h (intracellular staining) in the presence of medium or 2 μg/ml of Cdt. Surface CdtC was detected as previously described (Boesze-Battaglia et al., 2015). Briefly, cells were washed, exposed to normal mouse IgG (Zymed Labs; San Franscisco, CA) and then stained (30 min) for the CdtC subunit with anti-CdtC subunit mAb conjugated to AlexaFuor 488 (Molecular Probes; Eugene, OR). Following fixation with 2% paraformaldehyde the cells were analyzed by flow cytometry. Intracellular CdtB was detected after exposure of cells to toxin (or medium) and fixation with 2% formaldehyde for 30 min followed by permeabilization with 0.1% Triton X-100 in 0.1% sodium citrate and stained with anti-CdtB mAb conjugated to Alexafluor 488 (Molecular Probes).

### Statistical analysis

Mean ± standard error of the mean were calculated for replicate experiments. Significance was determined using a Student's *t*-test using SigmaPlot Software (Systat; San Jose, CA); a *P*-value of <0.05 was considered to be statistically significant.

## Results

**Figure 1:**
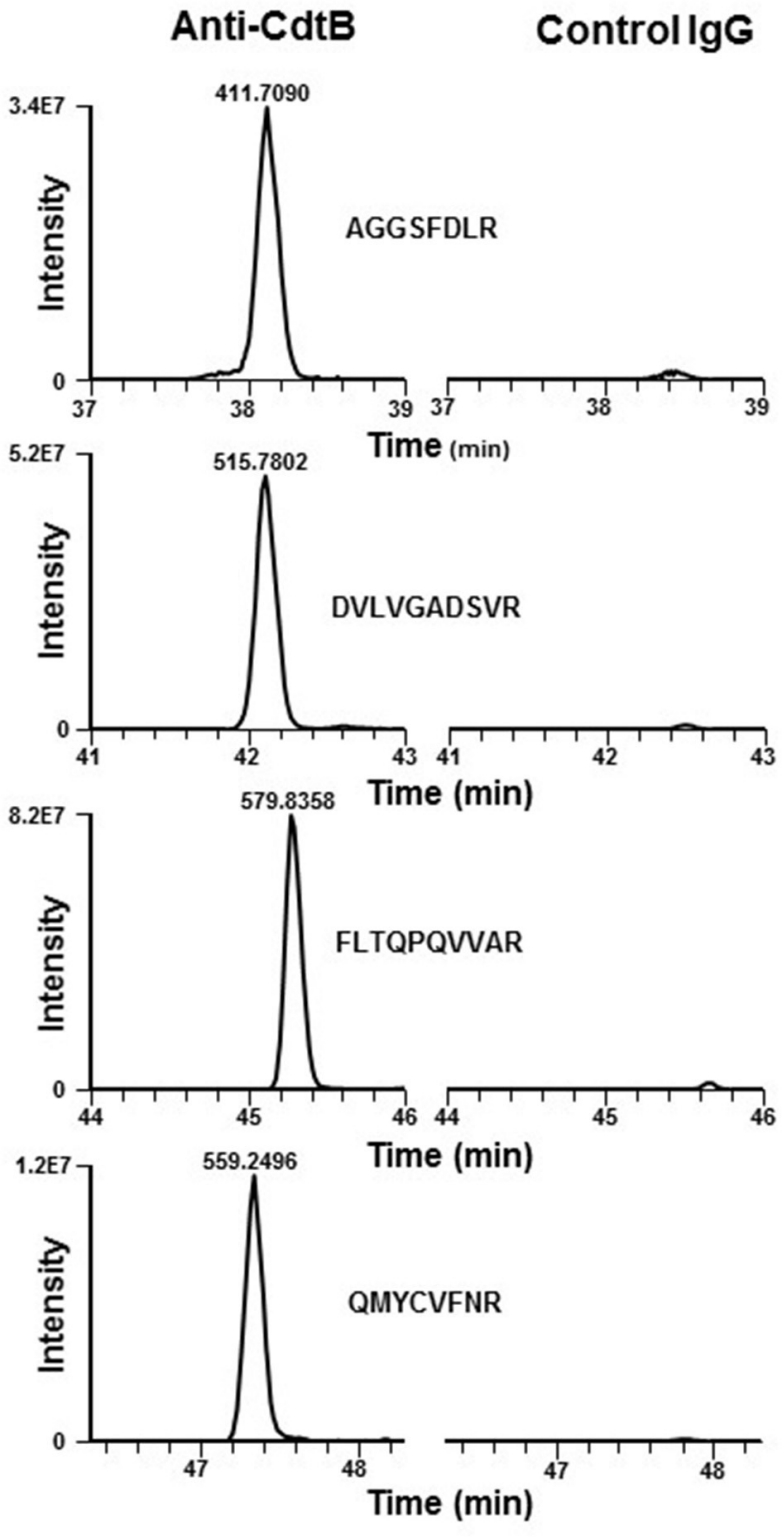
Extracted ion chromatograms of four representative doubly charged SYNGR2 peptides. Jurkat cells were treated with Cdt holotoxin as described in Materials and Methods for 1 h. Cell extracts were prepared and immunoprecipitated with immobilized anti-CdtB or isotype control IgG and the samples analyzed as described. The immunoprecipitates obtained with anti-CdtB mAb show strong signals for the peptides indicated; results are consistent with detection of MS/MS spectra for SYNGR2 peptides obtained exclusively with this mAb and were not detectable in the control immunoprecipitate.

In previous studies we have demonstrated that the ability of Cdt to intoxicate cells was dependent upon toxin association with cholesterol-rich membrane microdomains. Specifically, by employing laser confocal microscopy, we observed that all three Cdt subunits co-localized with GM1 ganglioside which is both enriched within and characteristic of membrane lipid rafts (Boesze-Battaglia et al., 2006). Western blot analysis of lipid raft fractions isolated as detergent resistant membranes (DRM) also demonstrated the presence of Cdt peptides within these cholesterol-rich regions. Proteomic analysis of an anti-CdtB immunoprecipitate obtained from toxin treated Jurkat cells identified the presence of a ubiquitously expressed protein, cellugyrin. Specifically, analysis of these samples by LC-MS/MS revealed 10 peptides consistent with detection of MS/MS spectra for SYNGR2 peptides (Table 1); chromatograms for four of these peptides are shown in Figure 1. We have now extended these observations to determine if cellugyrin interacts with CdtB in a manner that is critical to the internalization of this active subunit and for its ability to induce PI-3K signaling blockade, a requisite for toxin induced cell cycle arrest and apoptosis (Boesze-Battaglia et al., 2016; Scuron et al., 2016; Shenker et al., 2016a).

**Table 1:** Synaptogyrin-2 (SYNGR) peptides detected by LC-MS/MS.

**Sequence**	**Mass**	**Charge**	**^a^PEP**	**^b^Score**	**Intensity (anti-CdtB)**	**Intensity (control IgG)**
AAITFSFFSIFSWGVLASLAYQR	2581.3369	3	2.98E-06	65.184	6.28E+06	0
AGGSFDLR	821.4032	2	2.12E-02	68.224	2.06E+08	0
AGGSFDLRR	977.5043	2	4.10E-02	72.879	2.26E+08	0
DVLVGADSVR	1029.5455	2	5.06E-03	62.466	3.24E+08	0
DVLVGADSVRAAITFSFFSIFSWGVLASLAYQR	3592.8718	3, 4	4.22E-09	41.174	4.86E+06	0
FLTQPQVVAR	1157.6557	2	2.19E-03	99.815	6.30E+08	0
FLTQPQVVARAVCLVFALIVFSCIYGEGYSNAHESK	4073.0543	4	6.92E-19	59.748	9.30E+06	0
MESGAYGAAK	983.4382	2	6.73E-03	48.284	1.83E+07	0
QMYCVFNR	1116.4845	2	6.61E-05	101.28	1.41E+08	0
QMYCVFNRNEDACR	1861.7658	3	4.12E-04	87.083	2.11E+07	0

a *Posterior error probability of the identification*.

b Andromeda score

**Figure 2:**
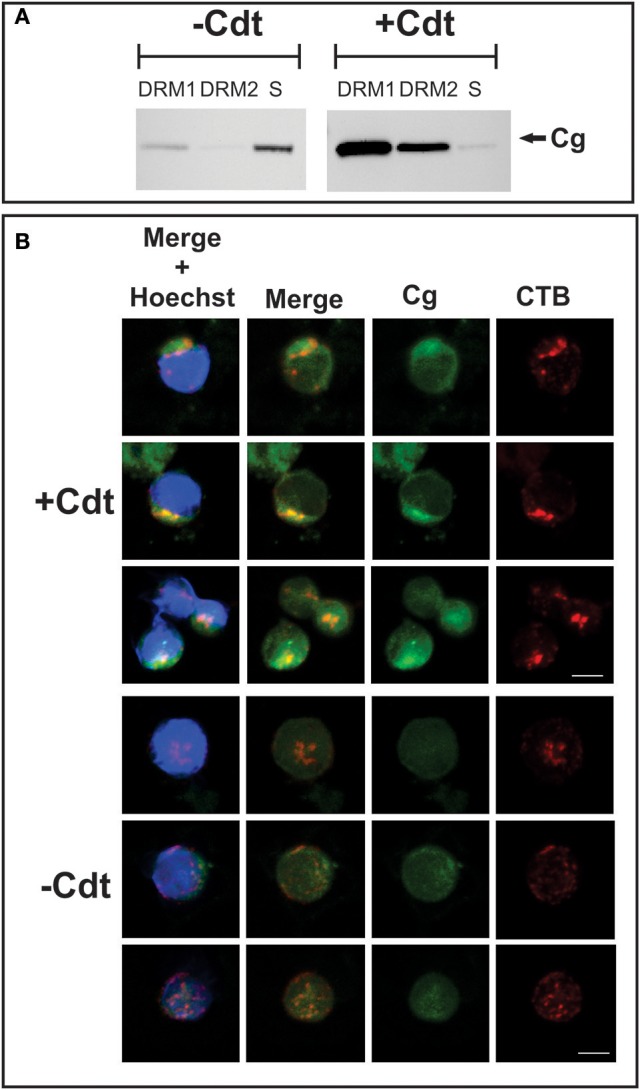
Translocation of cellugyrin to cholesterol rich micro domains. **(A)** Jurkat cells were treated with medium (−Cdt) or with 2 μg/ml Cdt for 2 h. Cells were harvested, washed, and cholesterol rich microdomains isolated as detergent resistant membranes (DRM) as described in Materials and Methods. Two DRM zones, designated DRM1 and DRM2, as well as a soluble fraction were obtained and further analyzed by Western blot for the presence of cellugyrin. Results are representative of three experiments. **(B)** Jurkat cells were treated with medium (−Cdt) or 1 μg/ml Cdt (+Cdt) for 1 h; cells were stained and fixed as described in Materials and Methods and analyzed by confocal microscopy. Maximum intensity projection of a 3 μm z-stack series is presented (3 cells/condition). For each image, fluorescence is shown for cellugyrin alone (green), lipid rafts using fluorescence of cholera toxin B (CTB; red) and merged images (yellow) with (blue) and without nuclear staining Results are representative of multiple fields and analysis of over 50 cells for each condition. Scale bar = 5 μm.

Cholesterol-rich microdomains were isolated as DRM fractions from both control (no toxin) and toxin-treated Jurkat cells after 2 h exposure to Cdt. Cells were disrupted by homogenization in ice-cold Triton X-100 and ultracentrifuged on a sucrose gradient; two distinct low buoyant density zones, designated DRM1 and DRM2, were obtained. We have previously demonstrated that these fractions contained membrane microdomains as evident by analysis for the presence of GM1 as well as cholesterol and phosphate content (Boesze-Battaglia et al., 2006). As shown in Figure 2A, Western blot analysis of these preparations demonstrate small quantities of cellugyrin in DRM1 isolated from control Jurkat cells and none in DRM2. In addition to DRM1 and DRM2 we also analyzed detergent soluble, non-lipid raft, material; the largest amount of cellugyrin was found in this fraction in control cells. The prevalence of cellugyrin in the latter soluble fraction is quite significant when accounting for the greater volume of this fraction. In contrast, Jurkat cells treated with Cdt for 2 h demonstrated a dramatic shift in the association of cellugyrin with lipid rafts as most of the cellugyrin was now found in the DRM1 and DRM2 zones. It should be noted that in earlier studies we observed that Cdt subunits A and C were primarily found in DRM1 and to a lessor extent in DRM2; detectable amounts of CdtC were observed within the soluble fraction. CdtB was found primarily in DRM1, but also in DRM2 and the soluble fraction (Boesze-Battaglia et al., 2006).

To further examine the translocation of cellugyrin to membrane microdomains, laser confocal microscopy was employed to assess Jurkat cells. Cells were treated with Cdt, or medium only, and then dual stained to identify cellugyrin with antibody conjugated to AlexaFluor (AF) 488 as well as GM1, a well-established marker for membrane rafts. To identify GM1, cells were stained with CTB conjugated to AF594; to visually identify microdomains, patches of GM1 were induced by treatment with anti-CTB antibody. As shown in Figure 2B, control Jurkat cells displayed low cytoplasmic immunofluorescence for cellugyrin with almost no detectable co-localization with CTB. In contrast, Jurkat cells treated with Cdt exhibited a shift in cellugyrin associated immunofluorescence as it now exhibits partial co-localization with CTB in close proximity to the plasma membrane.

**Figure 3:**
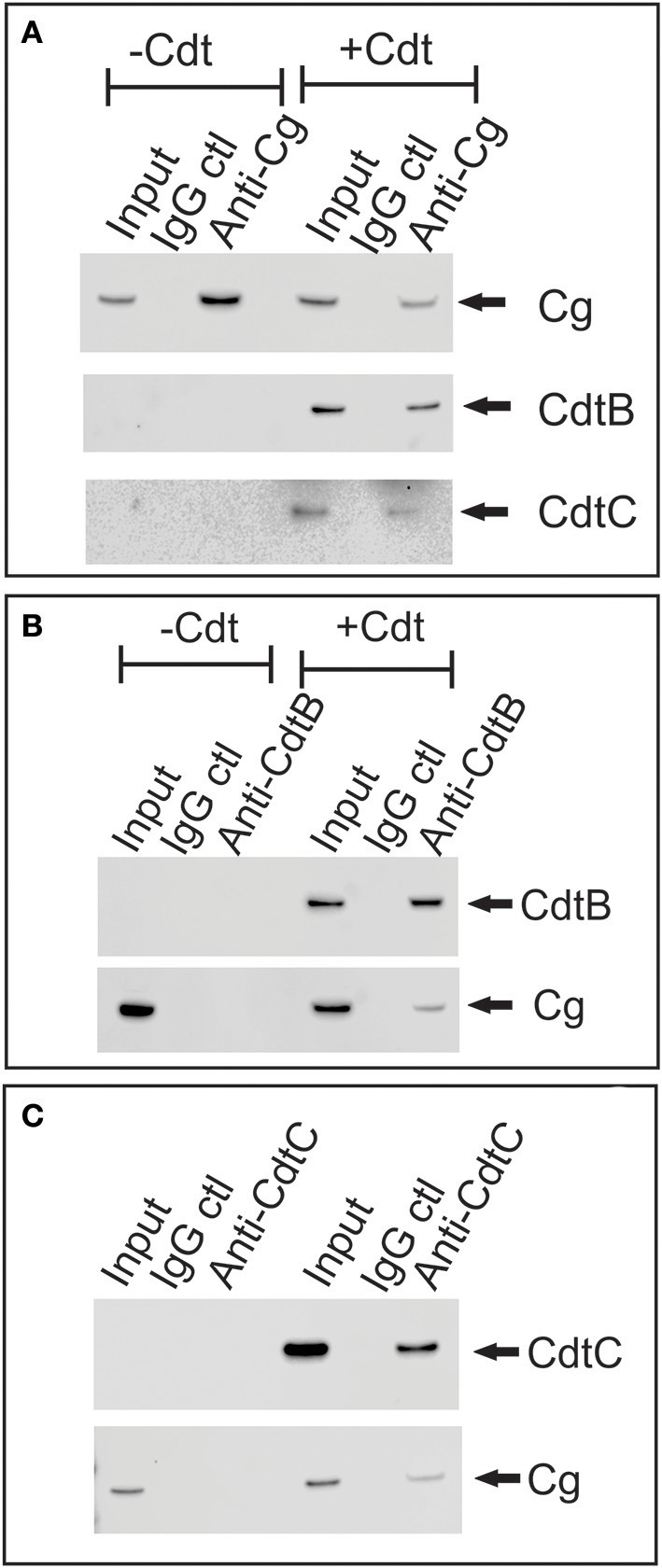
Immunoprecipitation of cellugyrin and Cdt subunits. Jurkat cells were treated with medium or Cdt (2 μg/ml) for 2 h and then washed and homogenized as described in Materials and Methods. **(A)** Shows the results of extracts immunoprecipitated with either immobilized control IgG or anti-cellugyrin antibody. The bound material was eluted and analyzed by Western blot for the presence of cellugyrin (Cg), CdtB or CdtC. **(B)** Shows the results of cell extracts obtained from similarly treated cells as above and immunoprecipitated with immobilized control IgG or anti-CdtB mAb. The bound material was eluted and analyzed by Western blot for the presence of CdtB and Cg. **(C)** Shows the results of cell extracts obtained from cells treated as described above and immunopreciptated with immobilized control IgG or anti-CdtC mAb. The bound material was eluted and analyzed by Western blot for the presence of CdtC and Cg. Results are representative of three experiments.

It should be noted that we were unable to utilize immunofluorescence in combination with confocal microscopy to demonstrate co-localization between CdtB and cellugyrin as immunostaining for each protein requires fixation and permeabilization protocols that were not compatible with one another. Therefore, we employed immunoprecipitation to demonstrate association between cellugyrin and Cdt subunits. As shown in Figure 3A, cell extracts obtained from control and Cdt-treated Jurkat cells were immunoprecipitated with immobilized anti-cellugyrin (Cg) Ab or control IgG; the immunoprecipitate was eluted, fractionated by SDS-PAGE and then analyzed by Western blot. Cellugyrin was observed in the immunoprecipitate from extracts derived from both control (medium only) and Cdt-treated cells. Additionally, the immunoprecipitate from toxin treated cells also contained CdtB and CdtC. CdtA was not detected in these preparations suggesting that this subunit remains associated with the membrane and is not internalized. Cellugyrin as well as Cdt subunits were not present in samples processed with immobilized control IgG.

**Figure 4:**
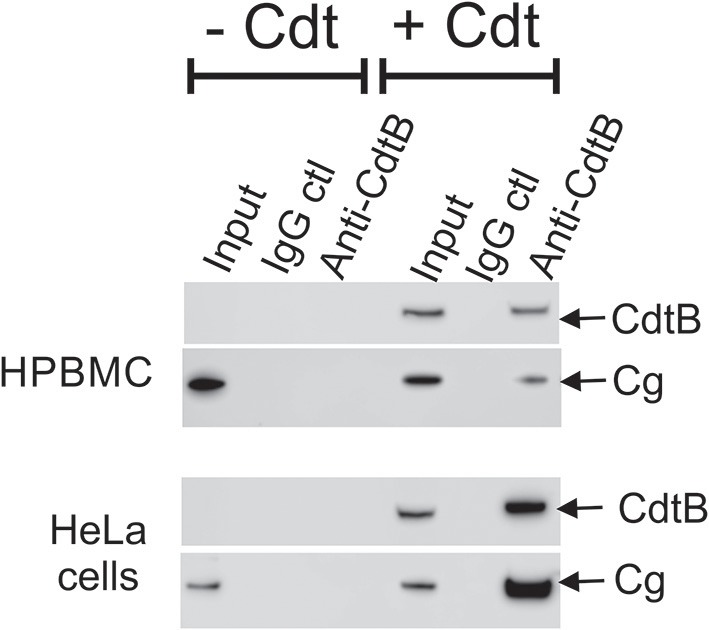
Immunoprecipitation of cellugyrin and CdtB in HPBMC and HeLa cells. HPBMC and HeLa cells were treated with medium or Cdt (2 μg/ml) for 2 h. Cell extracts were prepared as described in Materials and Methods and immunoprecipitated with immobilized control IgG or anti-CdtB mAb. The bound material was eluted and further analyzed by Western Blot for CdtB and cellugyrin. Results are representative of three experiments.

Western blot analyses of immunoprecipitates obtained using anti-Cdt subunit mAbs were performed. Jurkat cells were treated as above with either medium only or Cdt; cell extracts were then exposed to immobilized anti-CdtB or anti-CdtC mAb. As shown in Figure 3B, immunoprecipitation with immobilized anti-CdtB mAb not only pulled down CdtB, but also cellugyrin. Likewise, immunoprecipitates obtained with anti-CdtC mAb contained both CdtC and cellugyrin (Figure 3C). Control Ig did not immunoprecipitate either the toxin subunits or cellugyrin. Since cellugyrin is a ubiquitously expressed protein, we wanted to determine if it interacted with CdtB in other cells known to be susceptible to Cdt. Cell extracts were obtained from both HPBMC and HeLa cells treated with medium or Cdt and then processed with immobilized anti-CdtB or control Ig. In both instances, the immobilized anti-CdtB mAb immunopreciptated both CdtB and cellugyrin while neither was present with control Ig (Figure 4).

**Figure 5:**
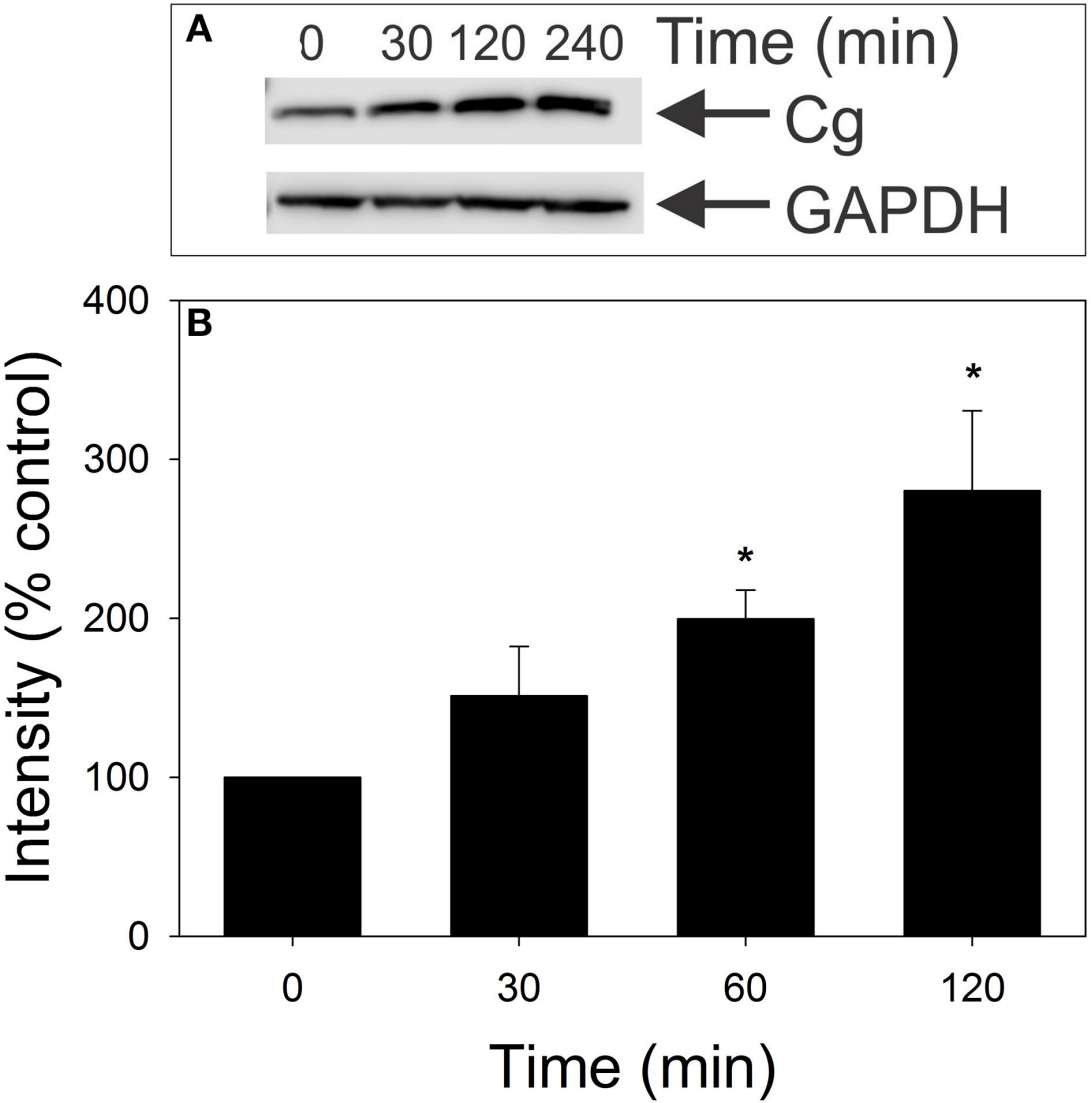
Effect of Cdt on cellugyrin levels in Jurkat cells. Jurkat cells were incubated in the presence of medium or 25 pg/ml Cdt for 0–120 min. Cells were harvested, homogenized and analyzed by Western blot for relative cellugyrin content. Blots were further analyzed by digital densitometry. Results of a representative blot are shown in **(A)** and the mean ± SEM of three experiments are shown in **(B)**; results are expressed as a percentage of the intensity observed in control cells. ^*^Indicates statistical significance (*p* < 0.05) when compared to untreated cells.

We next determined if, in addition to altering cellugyrin subcellular localization, exposure to Cdt also results in changes in its expression. Jurkat cells were treated with 25 pg/ml Cdt for varying periods of time and cell homogenates analyzed by Western blot for cellugyrin content. As shown in Figure 5, a 50% increase in cellugyrin protein levels was observed within 30 min of exposure to Cdt. Cellugyrin expression continued to increase at 60 and 120 min to 200 and 275% over that observed in control cells. It should be noted that we did not detect an increase in cellugyrin mRNA levels, but instead a slight decrease (Figure S1) suggesting that the observed increase in protein might be due to reduced degradation.

**Figure 6:**
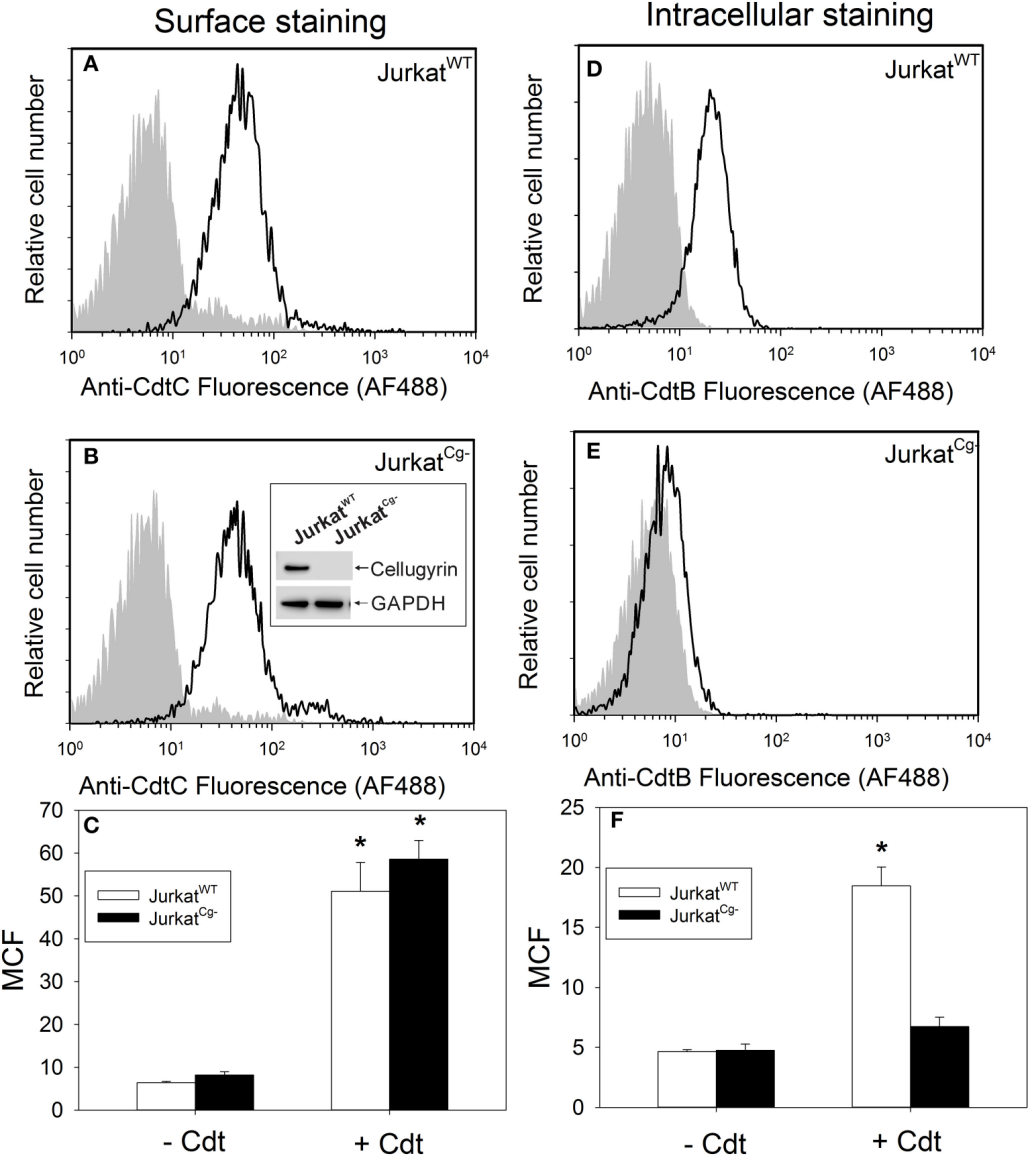
Cdt binding and CdtB internalization in Jurkat^WT^ cells vs. Jurkat^Cg−^ cells. Jurkat^WT^ and Jurkat^Cg−^ were first compared for their ability to bind Cdt (**A–C**). Cells were incubated for 60 min at 5°C with Cdt (2 μg/ml), washed and stained for the presence of cell surface associated Cdt using anti-CdtC mAb conjugated to AF488. Representative flow cytometric analysis for Cdt binding to Jurkat^WT^ is shown in panel A; solid line is the result obtained with Cdt-treated cells and the shaded curve represents cells exposed to medium alone. Cdt binding to Jurkat^Cg−^ cells is shown in **(B)**. Cells were generated as described in Materials and Methods; confirmation of their inability to express cellugyrin was demonstrated by Western blot as shown in **(B)** inset. Results from multiple experiments are shown in **(C)**; results are the MCF ± SEM obtained from three experiments. Internalization of CdtB in Jurkat^WT^ and Jurkat^Cg−^ cells was analyzed following exposure to Cdt (2 μg/ml) for 1 h at 37°C; cells were washed, fixed, permeabilized, and stained with anti-CdtB mAb conjugated to AF488. Representative results are shown for Jurkat^WT^ in **(D)** and for Jurkat^Cg−^ in **(E)**; solid line represents results obtained from cells treated with Cdt and the shaded curve from cells exposed to medium only. Results from multiple experiments are shown in **(F)**; results are the MCF ± SEM obtained from three experiments. ^*^Indicates statistical significance (*p* < 0.05) when compared to control (-Cdt) cells.

In order to advance our understanding of the biological significance of CdtB-cellugyrin interaction, we next determined if cellugyrin was critical for Cdt holotoxin association with cells, CdtB internalization, and/or toxicity. To achieve these goals, cellugyrin expression was eliminated by utilizing CRISPR/Cas9 gene editing of Jurkat cells. A cell line deficient in cellugyrin (Jurkat^Cg−^) was successfully prepared (Figure 6B inset). Jurkat^Cg−^ cells were first assessed for their ability to bind Cdt holotoxin; toxin binding to the cell surface was carried out at 5°C and monitored by immunofluorescence using anti-CdtC mAb conjugated to AF488. Representative association of Cdt with wildtype Jurkat (Jurkat^WT^) cells is shown in Figure 6A; Jurkat^WT^ cells consistently bound Cdt as these cells exhibited a mean channel fluorescence (MCF) of 51.1 ± 6.8 with anti-CdtC mAb (Figure 6C; open bars). Likewise, Jurkat^Cg−^ cells were also capable of binding comparable amounts of toxin as these cells exhibited a MCF of 58.6 ± 4.3 (Figures 6B,C). Control cells exposed to medium alone exhibited MCF of 6.4 (Jurkat^WT^) and 8.2 (Jurkat^Cg−^).

The dependence on cellugyrin for CdtB internalization was next evaluated by assessing immunofluorescent staining with anti-CdtB mAb following fixation and permeabilization in both Jurkat^Cg−^ and Jurkat^WT^ cells. Cells were exposed to Cdt for 1 h at 37° and then fixed, permeabilized and stained with anti-CdtB mAb conjugated to AF488. Figure 6D shows representative results of CdtB internalization in Jurkat^WT^ cells. CdtB was observed to be reproducibly internalized in Jurkat^WT^ cells as results from repetitive experiments exhibited a MCF of 18.5 ± 1.6 in toxin treated cells (Figure 6F); this compares to a MCF of 4.7 ± 0.2 in control cells. In contrast, Jurkat^Cg−^ cells did not exhibit internalization of CdtB as the MCF was 6.7 ± 0.8 in toxin treated cells vs. 4.8 ± 0.5 in control cells exposed to medium only (Figures 6E,F). This is a statistically significant reduction in Jurkat^Cg−^ cell associated fluorescence when compared to the MCF observed in Jurkat^WT^. It should be noted that in previous studies we have demonstrated that the immunofluorescence due to CdtB internalization observed in Jurkat^WT^ cells was both dependent upon permeabilization and temperature; cells not permeabilized or those incubated at 5°C failed to exhibit fluorescence when stained with anti-CdtB mAb (Shenker et al., 2014).

**Figure 7:**
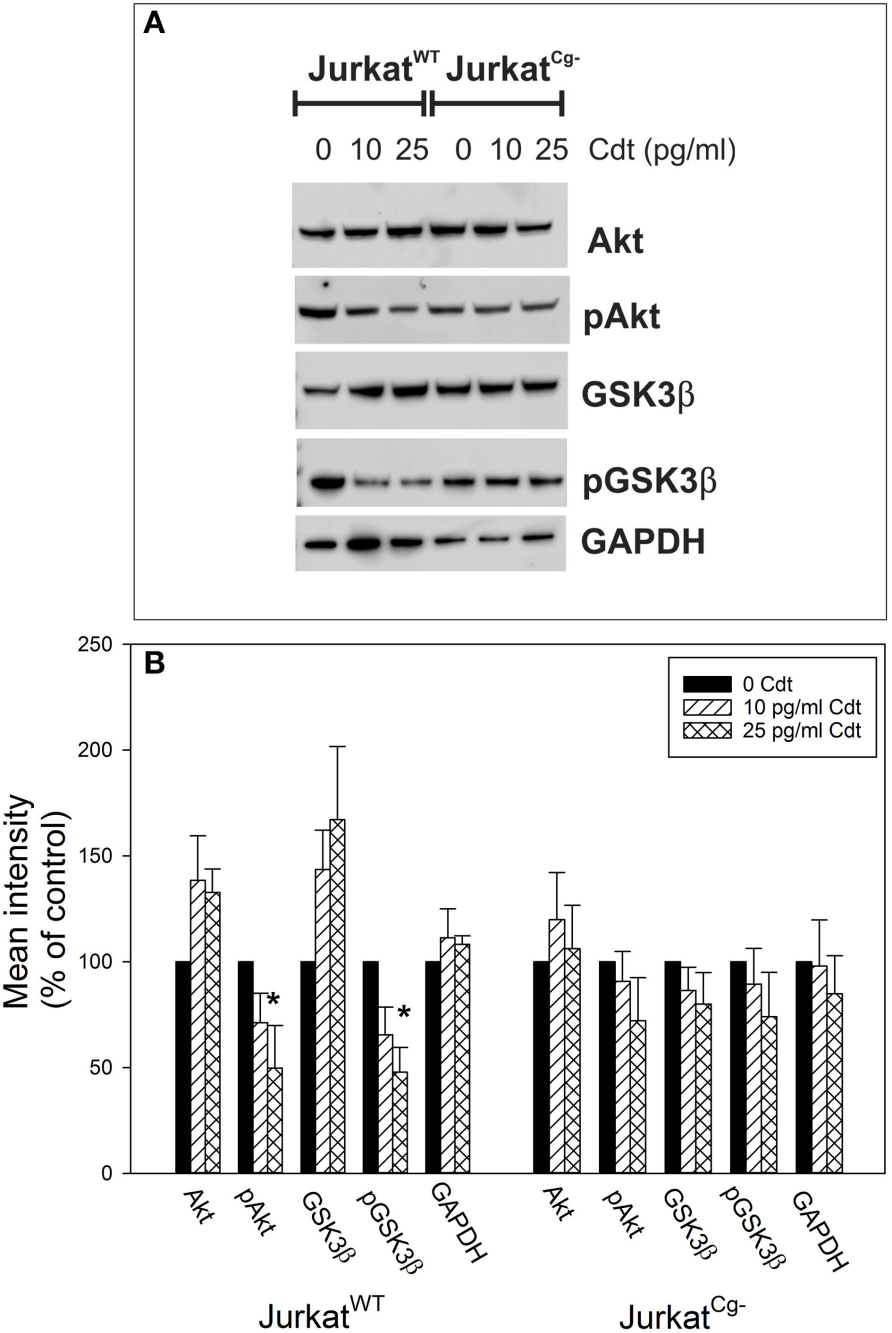
Comparison of the effects of Cdt on PI-3K signaling blockade in Jurkat^WT^ vs. Jurkat^Cg−^ cells. Jurkat^WT^ and Jurkat^Cg−^ cells were treated with 0–25 pg/ml Cdt for 2 h and then analyzed by Western blot for pAkt (S473), Akt, pGSK3β (S9), GSK3β, and GAPDH as a loading control. **(A)** Contains a representative Western blot showing the effect of Cdt on Akt and GSK3β phosphorylation. **(B)** Shows the results of Western blot analyses from three experiments; blots were analyzed by digital densitometry and are expressed as a percentage of the relative intensity of untreated control cells; mean ± S.E.M. for three experiments are plotted. ^*^Indicates statistical significance (*p* < 0.05) when compared to untreated control cells.

The final series of experiments focused on the requirement for cellugyrin in Cdt-mediated toxicity; specifically, Jurkat^Cg−^ cells were assessed for susceptibility to Cdt-induced PI-3K blockade, cell cycle arrest, and apoptosis. We have previously demonstrated that one of the earliest events following exposure to Cdt, and a requirement for downstream toxicity, is blockade of the PI-3K signaling pathway. In this regard, we have shown that the active Cdt subunit, CdtB, functions as a phosphatidylinositol 3, 4, 5-triphosphate (PIP3) phosphatase thereby depleting cells of the signaling lipid leading to a concomitant reduction in the phosphorylation status of downstream targets (Shenker et al., 2016a). To further confirm the failure of CdtB to be internalized in cellugyrin deficient cells, we next determined if treatment of Jurkat^Cg−^ cells with Cdt also failed to result in a change in the phosphorylation status of Akt and GSK3β. Jurkat^WT^ and Jurkat^Cg−^ cells were treated with 0–25 pg/ml Cdt for 2 h and the levels of Akt, pAkt, GSK3β, and pGSK3β were analyzed by Western blot. Figure 7A shows a representative Western blot and results from multiple experiments are shown in Figure 7B. Jurkat^WT^ cells exhibited reductions in Akt phosphorylation; pAkt (S473) was reduced to 71.1 ± 14.0 and 49.8 ± 20.1% of control values in the presence of 10 and 25 pg/ml Cdt, respectively. Total Akt levels were slightly elevated under these conditions: 38 and 33% above control values. In contrast, Jurkat^Cg−^ exhibited a small, but not statistically significant, reduction in pAkt in the presence of Cdt to 90.7 ± 14.1% (10 pg/ml) and 71.1 ± 20.4% (25 pg/ml). GSK3β is a downstream target for Akt; reductions in the phosphorylation status of Akt leads to a decrease in its kinase activity and a concomitant reduction in the downstream phosphorylation of GSK3β. Consistent with the reduction in pAkt, Jurkat^WT^ cells exhibit a reduction in pGSK3β (S9) to 65.5 ± 13.0% and 47.8 ± 11.7% of control levels in the presence of 10 and 25 pg/ml Cdt. Total GSK3β levels were observed to increase to 43.6 and 67% above levels observed in untreated Jurkat^WT^ cells. Jurkat^Cg−^ cells did not exhibit significant reductions in pGSK3β as toxin treated cells contained comparable amounts of this phosphorylated protein: 89.4 ± 16.9% (10 pg/ml Cdt) and 73.9 ± 21.0% of that observed in control cells.

**Figure 8:**
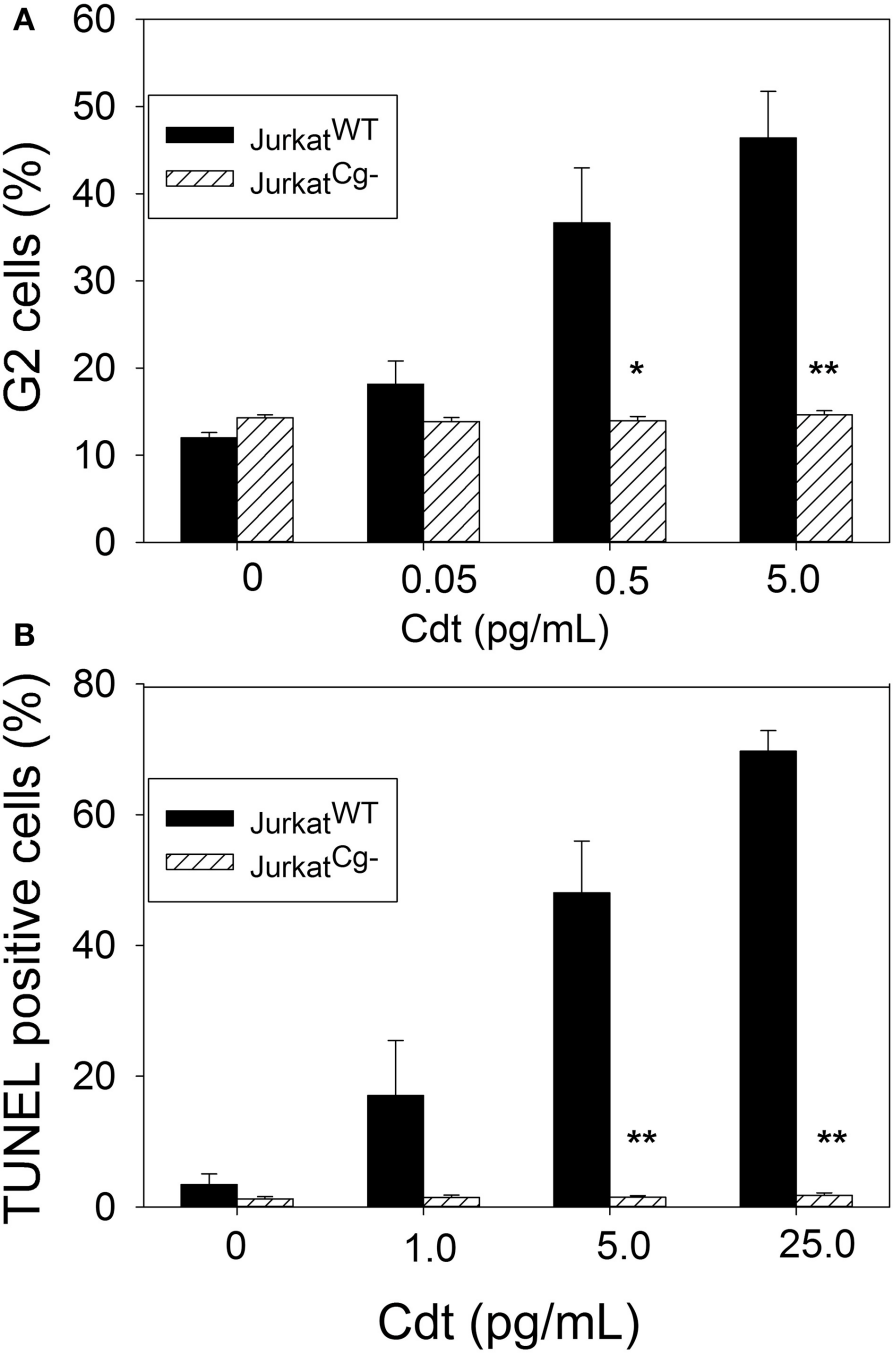
Comparative toxic effects of Cdt on Jurkat^WT^ and Jurkat^Cg−^ cells. **(A)** Shows the effect of Cdt on cell cycle arrest; Jurkat^WT^ and Jurkat^Cg−^ cells were incubated for 16 h in the presence of 0–5 pg/ml Cdt. Cells were stained with propidium iodide and cell cycle analysis performed using flow cytometry. The percentage of G2 cells is shown as a mean ± SEM for three experiments each performed in triplicate; solid bars represent Jurkat^WT^ cells and hashed bars Jurkat^Cg−^ cells. **(B)** Shows the effect of Cdt on apoptosis; cells were treated with 0–25 pg/ml Cdt for 48 h analyzed for DNA strand breaks using the TUNEL assay. Results are expressed as the mean percentage of TUNEL positive cells ± SEM for three experiments (); solid bars represent Jurkat^WT^ cells and hashed bars Jurkat^Cg−^ cells. ^*^Indicates statistical significance (*p* < 0.05) when compared to untreated cells; ^**^indicates statistical significance of *p* < 0.01.

Jurkat^Cg−^ cells were next assessed for their susceptibility to Cdt-induced cell cycle arrest. Cells were treated with Cdt for 16 h and cell cycle distribution was determined by measuring DNA content with propidium iodide and flow cytometry. As shown in Figure 8A, Jurkat^WT^ cells (solid bars) treated with Cdt exhibited cell cycle arrest; the cells were treated with 0.05–5 pg/ml Cdt and exhibited 18.2 ± 2.6 to 46.4 ± 5.4 percent cells in the G2/M phase of the cell cycle. Control cells treated with medium alone contained 12.0 ± 0.6 percent G2 cells. In contrast Jurkat^Cg−^ cells (hatched bars) did not exhibit cell cycle arrest as the percentage of G2/M cells did not increase when cells were treated with the same concentrations of Cdt. Exposure to 5 pg/ml Cdt resulted in 14.7 ± 0.5% G2/M cells; control cells incubated in medium alone contained 14.3 ± 0.3 percent G2/M cells.

Finally, to determine the requirement for cellugyrin in toxin-induced apoptosis, cells were treated with 1–25 pg/ml Cdt for 48 h and then analyzed for DNA strandbreaks using the TUNEL assay. Results are shown in Figure 8B and indicate that Jurkat^WT^ cells exhibited 3.5 ± 1.6, 17.0 ± 1.4, 48.0 ± 1.5, and 69.7 ± 1.8 percent apoptotic cells in the presence of 0, 1, 5, and 25 pg/ml Cdt, respectively. In contrast, Jurkat^Cg−^ cells exhibited no increase in the percentage of apoptotic cells over that observed in control cells; 1.8 ± 0.4 percent apoptotic cells was detected at the highest concentration of toxin (25 pg/ml) employed while control cells exhibited 1.3 ± 0.3 percent apoptotic cells.

## Discussion

It is well established that pathogens subvert host cell endocytic vesicles and retrograde trafficking to be effective. Once internalized, intracellular pathogens translocate a range of effector proteins to manipulate vesicle trafficking and signaling pathways thereby facilitating toxic and cytopathic events (reviewed in Personnic et al., 2016). Likewise, secreted bacterial toxins gain access to the cytosol by first binding to cell surface moieties that trigger endocytic uptake. Once internalized, vesicle trafficking provides access to retrograde transport to the TGN and eventually to intracellular target sites (Medina-Kauwe, 2007; Ahnert-Hilger et al., 2013; Harper et al., 2013; Backert and Tegtmeyer, 2017; Orrell et al., 2017). Thus, toxin-host cell interaction is facilitated by a common structure consisting of two units: the A unit representing the active (catalytic or enyzmatic) component which must be internalized and the B unit which is responsible for cell binding.

Most Cdt holotoxins utilize this AB_2_ structure where CdtB represents the internalized active unit and the binding unit is comprised of both CdtA and CdtC. CdtB internalization has been shown to occur by endocytic mechanisms dependent upon dynamin and involving clathrin coated pits (Cortes-Bratti et al., 2000; Thelestam and Frisan, 2004; Guerra et al., 2011; Guidi et al., 2013; Bielaszewska et al., 2017). These findings are consistent with the observations that Cdt binding and internalization involves CdtC recognition of cholesterol in the context of membrane microdomains (Boesze-Battaglia et al., 2006, 2009, 2015, 2016; Eshraghi et al., 2010; Zhou et al., 2012; Lai et al., 2013). Indeed, cholesterol rich membrane microdomains, often referred to as lipid rafts, are known to concentrate toxins and provide access to endocytic processes and signaling platforms (Cherukuri et al., 2001; Dykstra et al., 2003). The actual mechanism whereby CdtB is transported from cholesterol rich membrane microdomains to subcellular sites is controversial (Eshraghi et al., 2014) but likely involves the ERAD pathway.

In this study we demonstrate that exposure of Jurkat cells to Cdt leads to the translocation of the host cell protein, cellugyrin, from the cytosol to the plasma membrane in association with membrane lipid rafts. It is noteworthy that we have previously demonstrated that Cdt subunits also initially accumulate in the same region (Boesze-Battaglia et al., 2006, 2009, 2016). Toxin treated cells also exhibit increased levels of cellugyrin; however, RNA levels were decreased thereby suggesting that Cdt treatment may result in decreased cellugyrin degradation. Additionally, we demonstrate that shortly after exposure to toxin, the active Cdt subunit, CdtB, binds to a complex containing cellugyrin. These observations extend beyond an isolated observation in a lymphoid cell line as similar events were detected in primary HPBMC as well as HeLa cells which are commonly used as an experimental target for Cdt.

The significance of CdtB-cellugyrin interactions is exemplified by the observations that cells deficient in cellugyrin were able to bind Cdt holotoxin, but were unable to internalize CdtB. The inability to internalize CdtB is further corroborated by the finding that Jurkat^Cg−^ cells exhibited a concomitant resistance to CdtB-mediated toxicity. The toxin failed to induce a PI-3K signaling blockade, cell cycle arrest, and apoptosis (Shenker et al., 2007, 2016a,b; Scuron et al., 2016). It should be noted that this deficiency did not extend to other endocytic processes as Jurkat^Cg−^ cells did not exhibit alterations in transferrin receptor recycling, a process known to involve internalization of receptors associated with membrane lipid rafts (Figure S2). We now propose that cellugyrin is a key host cell protein critical to for Cdt toxicity by facilitating the internalization and subsequent translocation and retrograde transport of CdtB to key sites enriched in the signaling lipid, PIP3 (Shenker et al., 2007, 2016a,b; Boesze-Battaglia et al., 2016; Scuron et al., 2016). It should also be noted that these results are in agreement with those of Carette et al. (2011) who utilized insertional mutagenesis on a haploid background to disrupt gene function and identify critical genes for Cdt toxicity.

Cellugyrin is a member of a family of proteins known as synaptogyrins which contain four transmembrane regions with a tyrosine-phosphorylated tail (Janz and Sudhof, 1998); three synaptogyrin isoforms exist (Kedra et al., 1998). Synaptogyrins 1 and 3 are neuronal and are the most abundant protein in synaptic vesicles. It has been proposed that these synaptogyrins are critical to vesicle biogenesis, exocytosis, and endocytotic recycling as well as neurotransmission (Kedra et al., 1998; Hubner et al., 2002). In contrast, cellugyrin (synaptogyrin 2) is found in all tissue, except brain where it has been proposed to be a component of synaptic-like microvesicles (SLMVs) (Janz and Sudhof, 1998; Kupriyanova and Kandror, 2000; Belfort and Kandror, 2003; Kioumourtzoglou et al., 2015). Like synaptogyrin 1, cellugyrin has been shown to be critical for the biogenesis of cellugyrin containing SLMVs (Belfort et al., 2005). There is no information available to date that sheds light on the physiologic function of cellugyrin containing SLMVs in lymphoid or other cell types. Although it should be noted that Kupriyanaova and Kandror have shown that in adipose cells cytoplasmic cellugyrin positive SLMVs also contain Glut4; however, they do not localize to the plasma membrane following insulin treatment (Kupriyanova and Kandror, 2000). The authors proposed that these vesicles represent early sorting vesicles that are a component of the TGN. Further support that cellugyrin may exist in cytoplasmic vesicles comes from Chapel et al. (2013) who have suggested that this cellular protein is a lysosomal transporter protein. Of particular importance to our study is a recent report by Sun et al. (2016) who were studying Bunyavirus infection in mammalian cells and demonstrated that synaptogyrin-2 (cellugyrin) interacts with viral nonstructural proteins and together were transported into inclusion bodies “reconstructed from lipid droplets” during infection. They further reported that this translocation was critical to viral replication as silencing of cellugyrin expression reduced inclusion body formation and decreased virus titers.

It is becoming clear that cellugyrin and/or cellugyrin positive SLMVs may be a critical host target protein exploited by pathogens for the purposes of intracellular trafficking and retrograde transport of essential pathogen effector proteins to critical target sites. In this context, our current observations provide important insight into the earliest events that occur following the binding of Cdt holotoxin to target cells. There is agreement that in order for the active subunit, CdtB, to induce toxicity it must first gain access to intracellular compartments. Early studies on Cdt toxicity conducted by several investigators have suggested that the nucleus was the critical cellular compartment; these studies were conducted under a paradigm proposing that the critical events in Cdt toxicity were dependent upon CdtB's ability to function as a DNase (Elwell and Dreyfus, 2000; Cortes-Bratti et al., 2001; Frisan et al., 2002; Nesic et al., 2004; Thelestam and Frisan, 2004).

More recently, we have established a new paradigm for Cdt toxicity demonstrating that at least for *A. actinomycetemcomitans* Cdt, CdtB is a potent PIP3 phosphatase and further that this activity leads to PI-3K signaling blockade (Shenker et al., 2007, 2010, 2014, 2015, 2016a,b; Scuron et al., 2016). Thus, we propose that following internalization, CdtB must gain access to those intracellular sites containing enriched PIP3 pools. One such site is the cytosolic leaflet of plasma membranes particularly in regions associated with cholesterol enriched lipid rafts as these typically incorporate signaling platforms. Additional sites known to contain PIP3 include membrane bound subcellular compartments and, in particular, cytosolic structures such as, intracellular transport vesicles (Cockcroft and De Matteis, 2001; Simonsen et al., 2001; Di Paolo and De Camilli, 2006; Swanson, 2014). It is also noteworthy that phosphatidylinositols are known to play a role in governing the movement of these sorting vesicles. Interestingly, Xu et al. (2006) have shown cellugyrin positive SLMVs contain phosphatidylinositol 4-kinase; the authors suggest that polyphosphorylated derivatives of phosphatidylinositol may regulate vesicular traffic and protein recruitment. Thus, it is conceivable that CdtB's lipid phosphatase activity may enable the toxin subunit to re-direct and thereby re-purpose these vesicles to meet its needs of accessing PIP3 pools.

**Figure 9:**
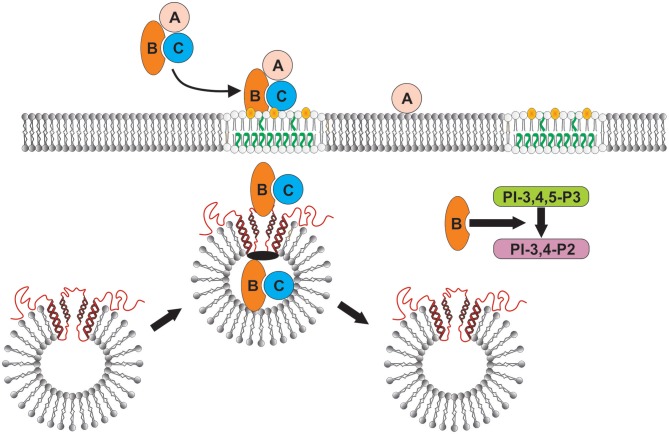
Schematic model showing proposed CdtB-cellugyrin interaction. Cdt holotoxin binds to cells via cholesterol in the context of membrane lipid rafts. CdtB binding and internalization is further dependent upon its ability to interact with cholesterol. As a result of exposure to Cdt, cellugyrin (shown in red) containing SLMVs translocate from cytosol to membrane lipid rafts. We propose that this translocation leads to the association of CdtB with the cellugyrin-containing SLMVs. This interaction may involve direct binding to cellugyrin either on extra- or intra-vesicular loops or indirect association via an unidentified binding partner (shown in black). We further propose that CdtB is transported via SLMVs to intracellular target sites; for example sites containing PIP3 pools where the enzymatically active CdtB subunit is released from SLMVs and is then able to degrade the signaling lipid resulting in PI-3K blockade and toxicity.

The latter observation may be critical to our findings that Cdt induces not only increased levels of cellugyrin, but also its translocation from the cytoplasm to the plasma membrane. Based on current understanding of the relationship between cellugyrin and SLMVs, these events likely lead to increased biogenesis of these vesicles and the likelihood of CdtB's hijacking them to facilitate its own translocation (Figure 9). In summary, we now propose that among the earliest critical events that occur following exposure of lymphocytes to *A. actinomycetemcomtans* Cdt involves the translocation of cellugyrin to cholesterol rich membrane microdomains and binding to CdtB (Figure 9). These interactions are essential as CdtB is unable to enter and translocate to intracellular sites in cells deficient in cellugyrin; these deficient cells exhibit a concomitant resistance to the toxic effects of Cdt which include PI-3K signaling pathway blockade, cell cycle arrest and apoptosis. We anticipate that future studies on CdtB-cellugyrin interactions will provide further insight into the role of cellugyrin positive SLMVs in the trafficking of CdtB to critical subcellular target sites. It is also likely that such studies will provide an understanding of the possible physiologic role(s) that cellugyrin and/or SLMVs play in cell homeostasis.

## Ethics statement

This study was carried out in accordance with the recommendations of the University of Pennsylvania's Office of Regulatory Affairs and the Institution Review Board with written informed consent from all subjects. All subjects gave written informed consent in accordance with the Declarationof Helsinki. The protocol was approved by the University of Pennsylvania Institutional Review Board.

## Author contributions

KB-B was intimately involved in the design and interpretation of all experiments and preparation of the manuscript. LW was instrumental in executing experiments and interpreting results. AD was instrumental in design, execution and interpretation of experiments relating to microscopy. KK was instrumental to experiments that relate to cellugyrin; specifically design of peptide for antibody production as well as, direction and interpretation of results and help in manuscript preparation. H-YT conducted proteomic analysis and provided insight that led to the identification of cellugyrin. BS was responsible for the overall study; he helped design experiments, interpret results particularly relating to toxin activity, role of cellugyrin and all aspects of data analysis; he significantly contributed to manuscript preparation.

### Conflict of interest statement

The authors declare that the research was conducted in the absence of any commercial or financial relationships that could be construed as a potential conflict of interest.
