# Supplementary figures and images for: Internalization of the Active Subunit of the Aggregatibacter actinomycetemcomitans Cytolethal Distending Toxin Is Dependent upon Cellugyrin (Synaptogyrin 2), a Host Cell Non-Neuronal Paralog of the Synaptic Vesicle Protein, Synaptogyrin 1
Source: Front Cell Infect Microbiol. 2017 Nov 14;7:469. doi: 10.3389/fcimb.2017.00469 (PMC5694546; doi:10.3389/fcimb.2017.00469)

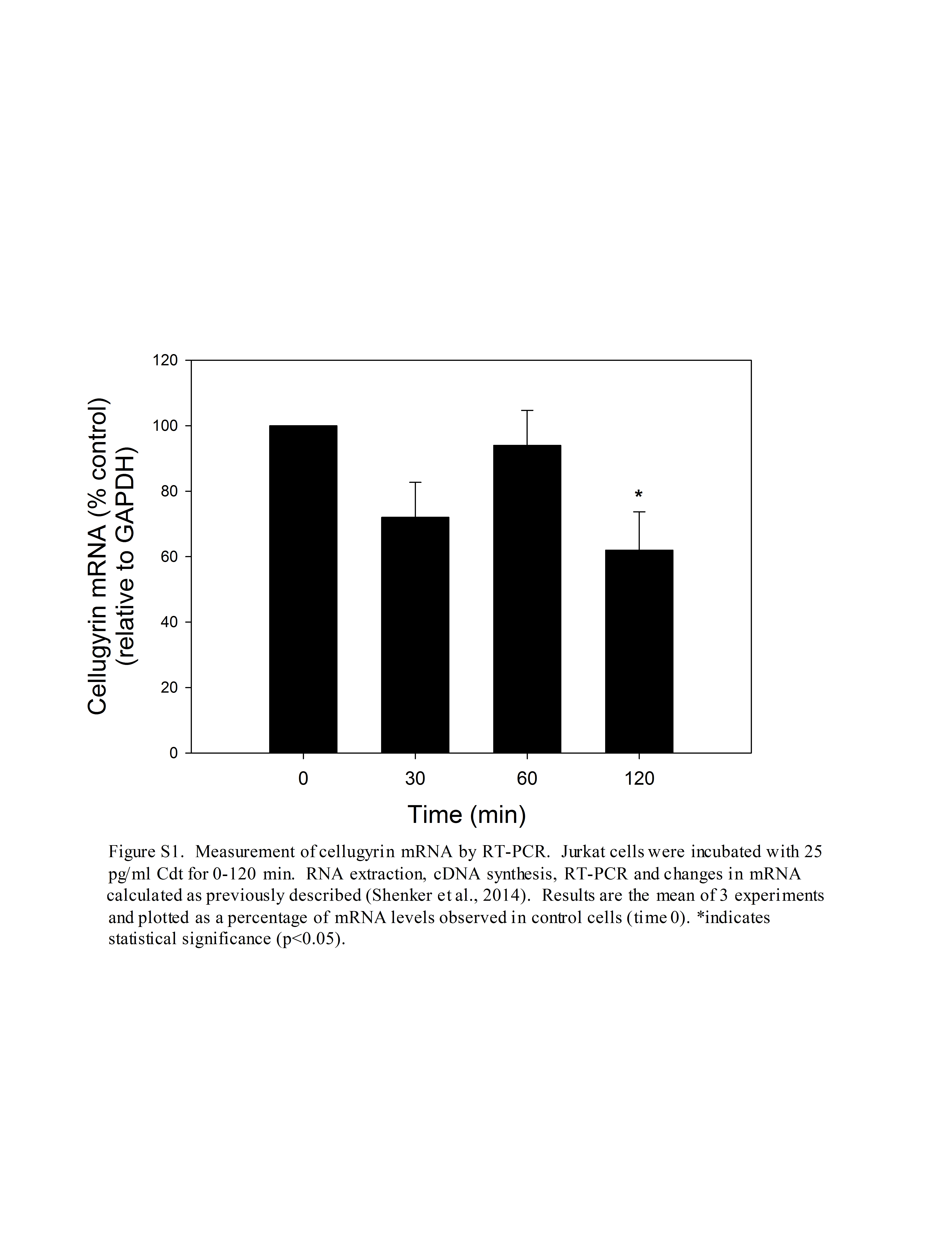

Supplement: Supplementary file 1 [file Image1.JPEG]

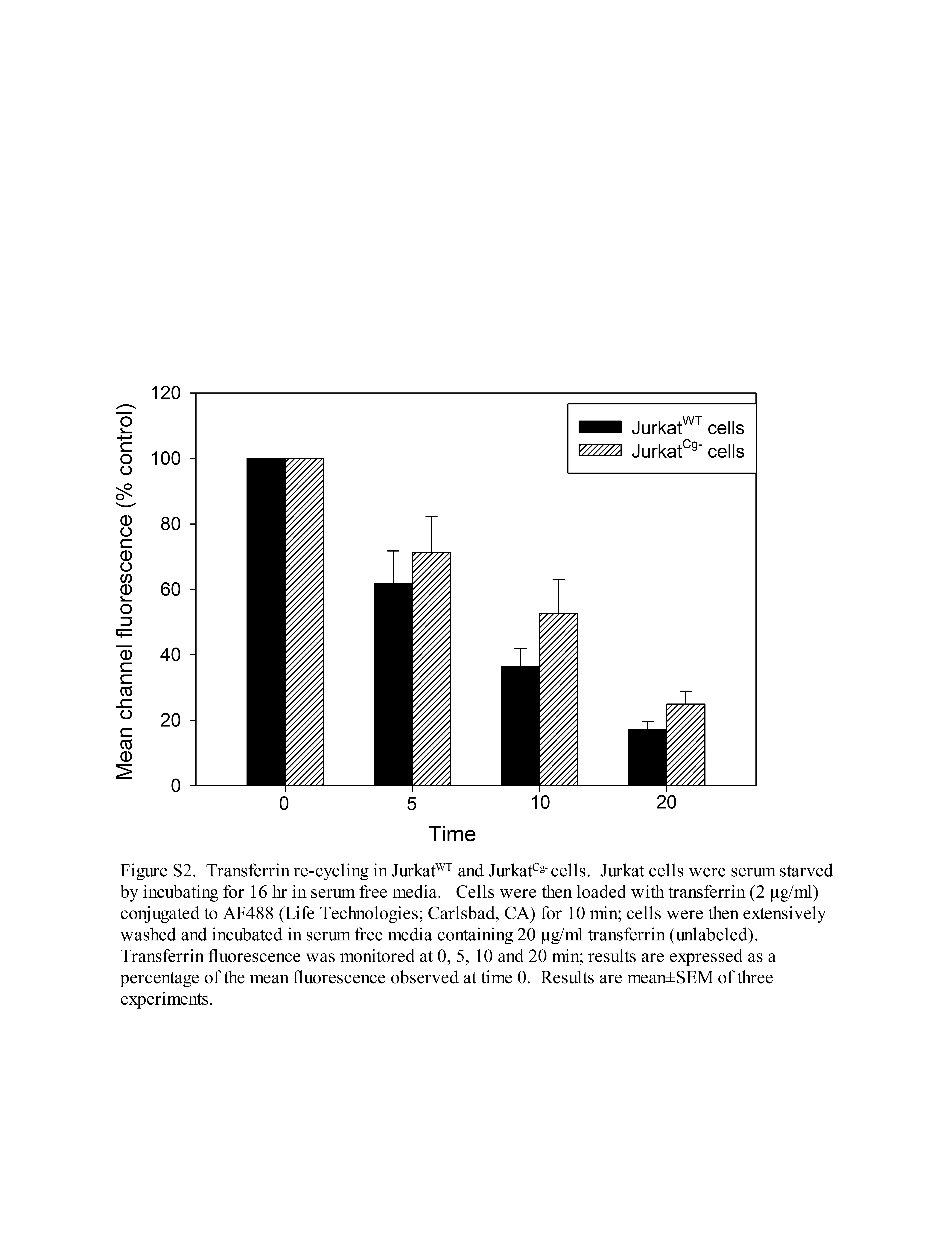

Supplement: Supplementary file 2 [file Image2.jpg]
